# Microbiological Retention on PTFE versus Silk Suture: A Quantitative Pilot Study in Third Molar Surgery

**DOI:** 10.3390/antibiotics12030562

**Published:** 2023-03-13

**Authors:** Stefano Parrini, Alessandro Bovicelli, Glauco Chisci

**Affiliations:** 1 Oral Surgery School, Dentistry and Dental Prosthodontics, Department of Medical Biotechnologies, University of Siena, 53100 Siena, Italy; 2 Independent Researcher, 53100 Siena, Italy

**Keywords:** suture, infection, PTFE, silk, surgery, third molar, extraction, sutureless, bacterial adhesion

## Abstract

**Background**: Mandibular third molar (M3M) removal and management of postoperative complications represent a common matter of interest in oral and maxillofacial surgery. This potential quantitative study assessed the ability of two types of surgical sutures, Silk and polytetrafluoroethylene polymer (PTFE), to carry aerobic and anaerobic bacteria on wounds after mandibular third molar surgery, with a collection of the stitches at the suture removal and study in the laboratory on the basis of colony-forming units. **Methods:** This prospective quantitative study sampled a total of 10 consecutive healthy patients for mandibular third molar surgery at the Oral Surgery School, Dentistry and Dental Prosthodontics, Department of Medical Biotechnologies, University of Siena, Siena, Italy. The mean age of the patients was 31 years (range 25–40 years), seven patients were male and three patients were female. Inclusion criteria were: presence of a partially impacted mandibular third molar. Exclusion criteria were: smoking and diabetes mellitus. Extraction of the mandibular third molar was performed under local anesthesia: after the third molar surgery, two sutures were applied on the surgical site distally to the second mandibular molar: one single 3/0 silk stitch; one single 3/0 PTFE stitch. No sutures were applied on the release incision. Sutures were removed after 7 days and were immediately conserved and sent to the laboratory to be rated on the basis of colony-forming units (CFUs). CFUs were evaluated and reported on GraphPad Prism and transformed into its base 10 logarithm. Data were analyzed with a non-parametric Wilcoxon test, and *p*-values < 0.05 were evaluated as statistically significant. **Results**: All the patients attended the suture removal date, and all the sutures were present in the site. None of the surgical sites presented dehiscence. No stitch loss was reported, and no patient reported mouth washing or tooth brushing in the surgery site. All interventions were uneventful and no major complications were reported after M3M surgery. Bacterial retention resulted as statistically greater in silk sutures rather than PTFE sutures, both in Brain Heart Infusion samples (*p* = 0.003) and Wilkins-Chalgren anaerobe samples (*p* = 0.002). **Conclusions**: We found the PTFE suture to be superior to the silk suture in a reduction in the bacterial biofilm in both aerobic and anaerobic evaluations after M3M surgery.

## 1. Introduction

Third molar removal is one of the most common interventions in oral and maxillofacial surgery current practice [1]. Hemostasis is vital for preventing blood clot loss and promoting healing after surgery, for both outpatients and hospitalized patients.

The extraction of mandibular third molars (M3M) leaves a decent size socket, which may be hard to heal via primary intention, due to the presence of wide space distal to the second molar, which can facilitate dehiscence and food impact. This highlights the importance of primary closure after M3M extraction. For this reason, hemostasis, represented by suture application, represents a key point following the extraction of the third molars, both for very complex extractions and easy flapless extractions.

Recently, in mandibular third molar surgery (M3M), the use of cyanoacrylate has been introduced as an alternative to the suture to perform a correct hemostasis, with interesting results [2]; however, the suture after M3M extraction, today, represents the most common procedure to achieve the hemostasis required for wound recovery, and this procedure is surely the most known and documented technique [3]. A variety of sutures have been used in dental extractions: silk has been the most common suture material in M3M surgery, and the main criticism it has received is regarding the bacterial biofilm formation over it [4]. With regards to suture technique in M3M surgery, international literature provides sutureless techniques, single suture techniques, different knotless suture techniques and the use of sutureless release incision techniques: each of these reported small advantages in terms of postoperative symptoms, time spent for suture and time saved after surgery [5,6,7,8,9]. Drainage inside the wound after M3M extraction and its retention until suture removal may have a role to reduce postoperative complications, too [10]. Among the complications that may appear after M3M surgery, alveolar osteitis and infection represent the most common and most feared adverse events [3]. Infection after M3M surgery may be due to many different causes, such as food infection and/or possible inflammation, caused by bacterial biofilm retention on the suture; all the suture materials can host or harbor bacterial biofilm formation to a variable degree.

The infection that can appear following a surgical extraction of a mandibular third molar may derive from the food impact that accumulates inside the wound or from the food that accumulates around the sutures. In fact, the type of suture can influence the amount of permitted food impact and/or bacterial plaque on the stitches. In the case of sutures that remain in the oral cavity for a prolonged time, the type of material also influences the entity of plaque that accumulates. Further, the use of the first intention suture technique or the second intention suture technique may have a role on food impact in the alveolus of the extracted third molar [11].

The oral biofilm formation on the sutures depends on the nature, type and material of the suture [12,13]. Asher et al., in their interesting randomized study, underlined this aspect, suggesting that the type of oral surgery did not significantly influence bacterial accumulation; also, periodontal diagnosis had little impact on bacterial counts. Interestingly, antibiotic administration after surgical treatment also had only minor effects on bacterial accumulation on the various sutures [14]. In fact, in this paper, the type of suture was superior to other aspects of surgery: the monofilamentous nylon sutures showed less microbial accumulation than the other tested materials that were all braided [14]. With regard to possible benefits of coated sutures versus non-coated sutures, Klaus et al. reported no advantages with the use of Triclosan-coated sutures [15]. Nadafpour et al. too underlined, in their research, regarding sutures in oral implantology, the superiority of nylon compared with others sutures [16].

Polytetrafluoroethylene (PTFE) is a material introduced in oral surgery with interesting biomechanical characteristics, such as smooth surface and no plastic memory [17]. Among the plausible characteristics of this material, there is a presumed possibility of reduced plaque formation and, therefore, reduced inflammation in the tissues around the extraction socket [17]. This benefit could be useful in operations, such as the extraction of the mandibular third molar, in which the operative site is characterized by a stagnation of saliva, food and the presence of plaque could easily evoke inflammation and infection of the pericoronal tissues during recovery.

For this reason, in this study, the authors hypothesized a possible advantage of PTFE versus a common old suture material, silk, in terms of bacterial retention to the stitches in partially impacted mandibular third molar surgery. The objective of this study is to evaluate the ability of two types of surgical sutures, silk and PTFE, to carry aerobic and anaerobic bacteria on wounds after mandibular third molar surgery with a collection of the stitches at the suture removal and a laboratory study on the basis of colony-forming units.

## 2. Materials and Methods

### 2.1. Patients

This is a prospective quantitative study of subjects scheduled for outpatient M3M surgery at the Oral Surgery School, Dentistry and Dental Prosthodontics, Department of Medical Biotechnologies, University of Siena, Siena, Italy. Inclusion criteria were: the IIB classification of Pell and Gregory, partially impacted mesio-angulated mandibular third molar, confirmed with a preoperative panoramic radiograph [18]. Exclusion criteria were: smoker patients and diagnosed diabetes mellitus.

Between September 2019 and December 2019, 10 patients with partially impacted mandibular third molar, mean age 31 years old (range 25–40 years), were included in this study: 7 patients were male and 3 patients were female. The study was conducted in accordance with the Declaration of Helsinki and approved by the Institutional Review Board. Informed consent was obtained from all subjects involved in the study. Written informed consent was obtained from the patients to publish this paper. For this study, the following sutures were used on each patient: 3/0 100% polytetrafluoroethylene polymer (PTFE) completely non-absorbable suture, white in color (DeOre, Negrar, Italy); 3/0 braided silk non-absorbable suture composed of an organic protein, black in color (Johnson & Johnson Medical N.V., Machelen, Belgium).

**Figure 1:**
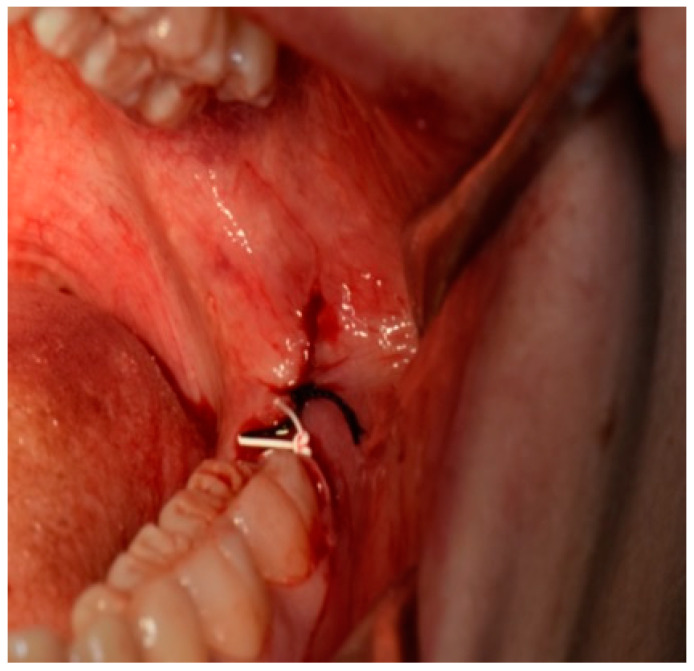
Image of PTFE and silk suture after M3M surgery placed distally to the second molar with a first intention closure technique.

All patients received oral hygiene performed by a professional oral hygienist 10 days before surgery and were educated on the correct home oral hygiene. All interventions and suture placements were performed by the same surgeon with more than 20 years of experience in oral surgery (S.P.): after local anesthesia with articaine 1:100.000, an incision was performed distally to the second mandibular molar with vestibular release incision. Further, a full-thickness mucoperiosteal flap was elevated; osteotomy around the mandibular third molar crown was then performed with bur, the tooth was dislocated with a straight lever and it was then completely removed with pliers. Accurate alveolar revision was then performed, and a saline lavage was performed inside the alveolus. After the tooth removal, two sutures with different materials were applied on the surgical site in order to obtain a first intention closure: one single stitch with 3/0 silk; one single stitch with 3/0 PTFE (Figure 1). Three knots per single stitch were used for both sutures: the order of the sutures was the same for all the patients involved in the study, with the PTFE placed distal to mandibular second molar and silk suture placed distal to PTFE suture.

Patients were educated to not brush on the surgical site and to avoid rinses with mouthwashes for seven days. The two sutures were removed after 7 days and were immediately inserted in two different 1 mL PBS/glycerol 10% solutions in sterile test tubes and sent to the laboratory for analysis. Postoperative drug administration for pain and swelling relief provided ibuprofen 600 mg every 12 h for all the patients.

### 2.2. Determination of Colony Forming Units

**Figure 2:**
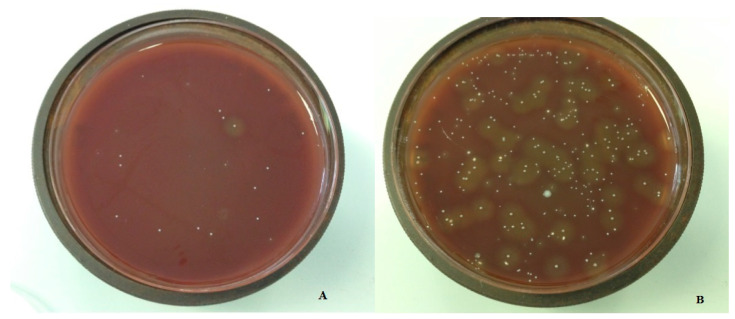
Bacterial growth around PTFE sutures (**A**); bacterial growth around silk sutures (**B**).

Laboratory procedures were performed by experienced staff, and two experienced lab technicians were involved in the calculation of CFUs. A blinding method was used for the laboratory transmission data: each sample was related with a number and no data of the nature of the suture was sent to the laboratory. For the data report from the laboratory, the information of the number was related to the nature of the suture. All the samples received sonic treatment for 10 s (Transsonic T460 Elma, Singen, Germany) to remove biofilm from the sutures and frozen at −80 °C. Adequate dilutions of every sample were prepared on plates for Brain Heart Infusion (BHI, Oxoid) agar and Wilkins-Chalgren anaerobe agar (WC, Oxoid) (Figure 2). WC plates were incubated at 36°C ± 1 °C in ambient air, while BHI plates were anaerobically incubated with Genbag (Biomerieux). Plates were evaluated every day for 72 h and colony forming units (CFUs) were calculated.

### 2.3. Statistical Analysis

CFUs were evaluated and reported on GraphPad Prism and transformed into its base 10 logarithm. Data were analyzed with a non-parametric Wilcoxon test, and *p*-values < 0.05 were evaluated as statistically significant.

## 3. Results

In total, 10 patients were operated on for M3M surgery. All the patients presented at the suture removal date, and all the sutures were present in the site after 7 days. None of the surgical sites presented dehiscence. No stitch loss was reported, and no patient reported mouth washing or tooth brushing on the site of the surgery. All interventions were uneventful and no major complications were reported after M3M surgery. Microbiological quantitative data are reported in Table 1.

**Figure 3:**
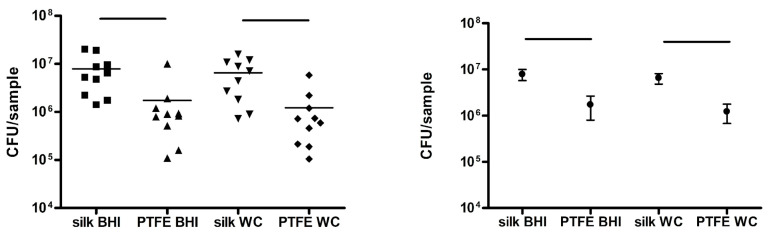
This graph on the left shows a comparison of CFUs of silk and PTFE sutures in BHI and WC; the population distribution is reported on the right.

Bacterial retention resulted as statistically greater in silk sutures rather than PTFE sutures, both in BHI samples (*p* = 0.003) and WC samples (*p* = 0.002). Logarithm graphs are reported in Figure 3.

On the basis of the laboratory results, a significantly greater accumulation of bacteria on the silk suture was reported on both samples (WC and BHI) compared to the PTFE suture, pointing out the superiority of PTFE in terms of bacteria retention 7 days after third molar surgery.

## 4. Discussion

Suture after third molar extraction is a great matter of interest in oral and maxillofacial surgery. The choice of suture technique after M3M surgery represents a topic that has great divergence among surgeons; in fact, this matter has been discussed many times in the literature: primary and secondary closure represents a challenging matter for many surgeons in order to influence the wound recovery and to obtain a better surgical outcome. While primary closure after M3M surgery appears to be more appealing for surgeons in order to protect the wound and the blood clot, to reduce the food impact in the wound and in order to reduce alveolar osteitis, secondary closure after M3M extraction appears to be more effective in reducing postoperative pain, facial swelling and trismus [19]; on the other hand, the proper role of suture and possible advantages of sutureless techniques after M3M surgery have been discussed too, reporting many controversial results [11,20]. The presence of suture after M3M surgery represents a vehicle for bacterial adhesion that elicits an inflammatory reaction [21]. This may be due to the needle injury, presence of suture around the alveolar pocket and/or the presence of bacterial plaque over the stitches. The development and presence of plaque and the possible development of inflammatory complications after M3M surgery may be annoying for both patients and clinicians [22,23]. In order to reduce postoperative infections, the use of local antibiotics inside the third molar socket at the end of the surgery has also been reported [24].

This current research in ten consecutive patients stated that the material of the suture plays a role in the bacterial retention as well as the formation of biofilm at the time of suture removal. The superior role of suture material over surgery and patient morbidity (periodontal disease) was reported in research from Asher et al. [14].

In this study, we used a first intention closure technique distal to the mandibular second molar in order to reduce the food impact in the surgical site; we hypothesized that this may somehow reduce the plaque accumulation variables that may occur in the case of different meals.

Many previous studies suggested a role of biofilm formation and bacterial adhesion in postoperative inflammatory complications. This eventuality is influenced by the physical, chemical and mechanical conformation of the suture materials [25]. Silk suture has been probably the most common material used in third molar surgery. Silk’s main advantages are physical resistance, maneuverability and low cost [26]. In M3M surgery, the physical advantages of silk are usually appreciated by both the surgeon and the patient; recovery after third molar surgery in the mandible is commonly influenced by chewing muscle activity, with significant stress on the suture around the alveolar pocket. In this study, we compared the bacterial growth on silk stitches versus PTFE stitches after mandibular third molar surgery. PTFE is a nonabsorbable material characterized by high biocompatibility, which, for many years, has been used for the production of vascular implants, heart valves and membranes for guided bone regeneration. Recently, expanded PTFE was introduced as a suture material in periodontal surgery and regenerative surgery [27,28,29].

In our study, PTFE showed the same physical resistance as silk suture; all the sutures were found on the surgical site after 7 days at the stitch removal stage and no stitch loss was reported. All the stitches were exposed to the same chewing muscle activity in the same patient. The “stitch loss” in third molar surgery represents an unfavorable point for the surgeon, a sign of high chewing activity, the sign of improper suture technique or the sign of an uncooperative patient. Commonly, in third molar suture studies, these data are lacking. Regarding the quantitative study on microbiological retention, PTFE showed statistically lower values than silk sutures in both aerobic and anaerobic samples after M3M extraction.

These data may appear to agree with previous studies regarding PTFE suture. In an in vitro study, Charbit et al. compared silk and PTFE, reporting the superiority of PTFE [30]. However, the main limitation of Charbit’s study was that it was an in vitro study, without the variability of the patient; our study, instead, evaluated sutures after M3M removal on ten consecutive patients. In a recent paper, Mahesh et al. studied bacterial adhesion on silk and PTFE after guided bone regeneration surgery, reporting good results for PTFE and silk, compared with vicryl and polyamide sutures; however, guided bone regeneration is rarely executed in the third molar site [31]. Further, in guided bone regeneration, the flap passivation technique is commonly executed, while this procedure is uncommon in M3M surgery. Flap passivation in guided bone regeneration is a technique that allows for complete coverage of the bone graft; this aspect cannot be compared with the common secondary or primary closure in M3M surgery [32].

Few papers in the literature have compared silk and PTFE. Scarano et al. reported an interesting study of sutures with silk versus PTFE in sinus lift surgery, reporting reduced bacteria colonization on PTFE and suggesting that the multifilament structure of silk favors a greater bacterial adherence, proliferation and persistence, so monofilament and e-PTFE suture should be preferred in oral surgery [33].

The main difference between the present study and the research of Scarano is the examined operation. While sinus lift is an operation with first intention suture and usually without local infection, the operation of extraction of M3Ms is commonly related to a contaminated surgical field, with bacteria from the pericoronitis surrounding the tooth and an increased stagnation of saliva due to the position in the oral cavity [33]. Regardless of the differences in operation, the results are similar and suggest better results with PTFE in oral surgery.

Pons-Vicente et al., in their study, compared silk- and teflon-coated polyester sutures (similar to PTFE) in implant surgery and reported a more pronounced plaque accumulation for silk sutures, without a statistical difference [34]; further, Pons-Vicente et al. reported that silk was reported as less comfortable for the patient than teflon-coated polyester sutures [34]. Compared to our study, this research reports results from a “clean” surgery, as well as Scarano et al., compared to the M3M extraction reported in our study. On the basis of our literature review, this is the first article that reports examinations of this suture with regard to M3M surgery with a quantitative bacterial growth evaluation. This lack of PTFE studies after M3M surgery may be due to an excessive trust on this suture or due to a higher cost, compared to the silk. The cost of suture is a parameter to take into account in oral surgery. Waite and Cherala, in their study, reported the outcome of 366 impacted third molar patients treated with a sutureless technique, opening the research to the sutureless technique in M3M surgery. However, cost-guided surgery did not show the expected relevant outcomes [35]. The use of a single filament suture was recently supported by the literature for prolonged recovery time [27,36]. However, the possibility to reduce plaque retention on a suture after M3M surgery represents a matter of interest in order to reduce postoperative complications. In our article, we reported positive outcomes for the PTFE suture after partially impacted mandibular third molar extraction. We conducted a study on bacterial growth on sutures with aerobic and anaerobic inoculation. The main limits of our study are the small number of patients, but with a complete follow-up of all patients, and the placement of the two stitches on the same side. In this study the split mouth was not performed but both the sutures were applied on the same surgical site; this reduces possible variables among two sides that the patient may have (e.g., tooth brushing, absence of teeth and a favorite chewing side). Further, we used the same order of the suture placement in all patients; a different order of the sutures among the patients could influence the results. If the stitch distal to the first stitch could be more exposed in the oral cavity and not “protected” by the second molar, the stitch near the second molar could be more influenced by the plaque arising on the distal surface of the tooth, but the results reported less bacteria on the PTFE stitch near the tooth. In the end, BHI is a nonselective agar for bacteria and the results could have been influenced by this aspect, albeit our results are overlapping the results of the previous research on PTFE sutures in oral surgery. For the limits we reported and the pilot nature of this study, we advocate further studies on this matter in order to evaluate the advantages of PTFE and comparison with nylon suture in mandibular third molar surgery. The superiority of PTFE over silk in terms of bacterial retention may be due to the structure of multifilament silk, which presents a structure with a hospitable niche for bacterial growth and proliferation. The microorganisms present in the multifilament are resistant to immune response, antimicrobial therapy and may produce a biofilm that increases microbial persistence.

## 5. Conclusions

We found the PTFE suture to be superior to the silk suture in the retention of bacterial biofilm in both aerobic and anaerobic evaluations after partially impacted mandibular third molar extractions; for this reason, on the basis of our pilot study, we suggest that the first choice of suture should be PTFE in third molar surgery.

## Figures and Tables

**Table 1:** Microbiological quantitative report of the stitches after removal at the suture removal 7 days after mandibular third molar surgery. * values expressed as base 10 logarithm measured as colony forming units.

Patients	BHI Silk *	BHI PTFE *	WC Silk *	WC PTFE *
1	6.72	5.90	7.20	5.77
2	6.34	5.20	5.86	5.33
3	5.88	6.05	6.43	5.02
4	6.67	5.92	6.64	5.86
5	6.94	5.04	6.94	5.28
6	6.98	5.72	7.08	6.34
7	7.30	7.10	7.03	6.76
8	7.28	6.28	6.85	6.08
9	6.15	5.96	5.95	5.66
10	6.80	5.97	6.26	5.87

## Data Availability

No data available.
